# Analysis of 3D Scan Measurement Distribution with Application to a Multi-Beam Lidar on a Rotating Platform

**DOI:** 10.3390/s18020395

**Published:** 2018-01-30

**Authors:** Jesús Morales, Victoria Plaza-Leiva, Anthony Mandow, Jose Antonio Gomez-Ruiz, Javier Serón, Alfonso García-Cerezo

**Affiliations:** Robotics and Mechatronics Lab, Andalucía Tech, Universidad de Málaga, 29071 Málaga, Spain; jesus.morales@uma.es (J.M.); victoriaplaza@uma.es (V.P.-L.); amandow@uma.es (A.M.); jseron@uma.es (J.S.); ajgarcia@uma.es (A.G.-C.)

**Keywords:** 3D laser scanner, multi-beam lidar, spatial data analysis, tilting mechanism, 3D scan pattern analysis

## Abstract

Multi-beam lidar (MBL) rangefinders are becoming increasingly compact, light, and accessible 3D sensors, but they offer limited vertical resolution and field of view. The addition of a degree-of-freedom to build a rotating multi-beam lidar (RMBL) has the potential to become a common solution for affordable rapid full-3D high resolution scans. However, the overlapping of multiple-beams caused by rotation yields scanning patterns that are more complex than in rotating single beam lidar (RSBL). In this paper, we propose a simulation-based methodology to analyze 3D scanning patterns which is applied to investigate the scan measurement distribution produced by the RMBL configuration. With this purpose, novel contributions include: (i) the adaption of a recent spherical reformulation of Ripley’s *K* function to assess 3D sensor data distribution on a hollow sphere simulation; (ii) a comparison, both qualitative and quantitative, between scan patterns produced by an ideal RMBL based on a Velodyne VLP-16 (Puck) and those of other 3D scan alternatives (i.e., rotating 2D lidar and MBL); and (iii) a new RMBL implementation consisting of a portable tilting platform for VLP-16 scanners, which is presented as a case study for measurement distribution analysis as well as for the discussion of actual scans from representative environments. Results indicate that despite the particular sampling patterns given by a RMBL, its homogeneity even improves that of an equivalent RSBL.

## 1. Introduction

There is a growing interest in portable and affordable three-dimensional (3D) lidar systems for new applications that can benefit from accurate and speedy range measurements, such as progress tracking in construction sites [1], precision agriculture [2], medical imaging [3], intelligent surveillance [4], or textile tailoring [5]. An alternative to high-end terrestrial scanners, such as those used for digital terrain modeling or forest inventory [6], is to obtain dense 3D data by aggregating multiple views from a less expensive sensor. At present, the most common approach to build compact 3D devices from low-cost sensors is tilting or spinning a 2D rangefinder by mounting it onto a one degree-of-freedom (DOF) rotation mechanism. Many examples of this type of customized rotating single-beam lidar (RSBL), mainly from the robotics community, can be found in the literature (e.g., [7,8,9,10]).

In the last few years, automotive applications have fostered an active market for new compact and cost-effective multi-beam lidar (MBL) rangefinders, such as those developed by Velodyne (Morgan Hill, CA, USA) [11]. As opposed to single-beam 2D sensors, in multi-beam rangefinders the rotating mirror is replaced by a spinning structure that holds a number of independent laser transceivers to scan different elevation angles within a given vertical field of view (FOV). This arrangement favors high data rates when compared to other 3D lidar configurations, but commercial MBLs offer limited vertical resolution and have to be rotated in order to produce a complete spherical FOV [12].

As MBLs are becoming increasingly popular and affordable, customized rotating multi-beam lidars (RMBL) built by adding one DOF to a commercial MBL may arguably become a common solution to obtain affordable rapid full-3D high resolution scans in the near future. This idea is supported by the introduction of customized RMBLs in recent research works [12,13,14].

The analysis of the scan measurement distribution, which can be appreciated qualitatively by simulating measured points on a hollow sphere [15], is crucial to exploit the potential capabilities of a 3D scanner. One major difference of RMBLs with respect to other 3D lidar configurations is the complexity of the resulting scanning pattern. Contrary to RSBLs, the additional DOF in RMBLs causes multiple scan beams to overlap, so measurements are not distributed with constant horizontal and vertical angular resolutions. Thus, the adoption of spatial data distribution indicators, such as Ripley’s *K* function [16], can be useful to analyze complex 3D scanning patterns from new rangefinder configurations and devices, not only qualitatively but also quantitatively.

In this paper, we investigate the promising RMBL configuration by proposing a simulation-based methodology to analyze the particular scanning patterns resulting from the addition of a DOF to a MBL. With this purpose, novel contributions include: (i) the adaption of a spherical formulation of the *K* function [17] to assess 3D sensor data distribution on a simulated hollow sphere; (ii) a comparison, both qualitative and quantitative, of general scan patterns produced by an ideal VLP-16-based RMBL with other 3D scan alternatives (i.e., RSBL and MBL); and (iii) a new RMBL implementation consisting of a portable tilting platform for VLP-16 scanners, which is presented as a case study for measurement distribution analysis as well as for the discussion of actual scans from representative environments.

The rest of the paper is organized as follows. Section 2 reviews related work. Section 3 defines an ideal RMBL based on Velodyne MBL specifications. Section 4 presents the analysis methodology. Section 5 analyzes RMBL scan patterns and offers comparisons with alternative 3D configurations. Section 6 describes a new VLP-16-based tilting platform, which is used to scan three representative environments that are discussed in Section 7. Finally, Section 8 offers the conclusions.

## 2. Related Work

This section reviews related work. First, it offers an overview of affordable 3D lidar sensors. Then, it discusses methods used to evaluate 3D scan data.

### 2.1. Affordable Solutions for 3D Lidar Sensors

For more than fifteen years, the most common alternative to high-end 3D lidar sensors has been to build customized devices where a less expensive off-the-shelf rangefinder is rotated by a servo-drive mechanism. Table 1 offers a chronology of representative works in which customized 3D lidar sensors have been proposed, analyzed or applied to particular problems. Most of these works correspond to RSBLs, whose aim is to achieve 3D point clouds by rotating a 2D sensor. The first works were based mainly on the 180° 2D Sick LMS200 (Waldkirch, Germany) scanner, which was later substituted by lighter and more compact devices such as the 270° Hokuyo UTM-30LX (Osaka, Japan).

More recently, MBL rangefinders commercialized by Velodyne are becoming increasingly popular and affordable. MBLs can be considered as a hybrid between 2D and 3D scanners, as they consist on a spinning structure that holds a number of independent laser transceivers to scan planes with different fixed elevation angles within a limited vertical FOV [11].

The Velodyne VLP-16 and HDL-32 rangefinders are representative examples of the most affordable end of commercial multi-beam sensors. Their major specifications are summarized in Table 2. The VLP-16 (or Puck) [33] is a more compact and lightweight device. From an operational standpoint, the major differences between these two sensors lie in the number of laser transceivers and the vertical FOV: The VLP-16 scanner has 16 individual laser/detectors arranged in a $30^{\circ }$ FOV, which yields a vertical resolution of $2.0^{\circ }$ with a data rate of 300,000 points/s, whereas the HDL-32 has 32 transducers within a FOV of $41.3^{\circ }$ with a vertical resolution of $1.33^{\circ }$ and a correspondingly higher data rate. In contrast with the VLP-16, whose FOV is symmetrical with respect to the horizontal plane, the FOV of the HDL-32 has a downward shift. Furthermore, the increased vertical resolution of the HDL-32 has a significant impact in the cost of the sensor, which is substantially more expensive than the VLP-16. These features have favored the adoption of MBLs in mobile applications, where dynamic point cloud registration along the vehicle’s path compensates for device limitations in vertical resolution and FOV [34]. Nevertheless, MBLs have to be rotated to yield a complete spherical FOV [12].

Given the compact size, high data rate, and decreasing cost of MBLs, customized 3D sensors built as rotating multi-beam lidars (RMBL) have the potential to become a common solution to obtain affordable rapid full-3D high resolution scans in the near future. The emergence of the RMBL configuration is indicated by recent examples shown in Table 1. For instance, the rotating 2D Hokuyo UTM-30LX-EW used by the Momaro robot in the DARPA Robotics Challenge of 2015 [10] has recently been replaced by a rotating Velodyne VLP-16 [14]. Moreover, Neumann et al. [13] built an RMBL based on a high-end 15 kg Velodyne HDL-64E to map underground mines from a wheeled robot. In a later work, these authors have developed a rotating platform based on a Velodyne VLP-16 that also includes a 2D Hokuyo and other sensors [12]. Moreover, a tilting HDL-64E has been used in [31] for robotic tunnel mapping.

### 2.2. Evaluation of Scan Data

Most of the works addressing 3D scan data quality have focused on calibration methods to compensate for inaccurate intrinsic parameters with evaluation of the accuracy, repeatability and stability of 3D measurements. This is particularly necessary for customized devices, which are prone to construction misalignments [23]. In particular, intrinsic calibration of RSBLs has been extensively treated in the literature (e.g., [23,24,32]). Besides, some recent works have proposed calibration methods to improve factory parameters in MBLs [35,36]. Temporal instability of MBL measurements poses another relevant calibration problem [37]. In this sense, temporal variability of calibration parameters and performance deviations between individual beams have been evaluated for the Velodyne VLP-16 [38]. As for the RMBL configuration, to our knowledge, no specific calibration methods have been proposed yet.

Not so many works have explicitly addressed the analysis of 3D lidar data in terms of the resulting measurement distribution, which is fundamental to exploit the potential capabilities of a particular sensor/DOF combination for a given application [12,15]. Wulf and Wagner [15] analyzed the scanning patterns resulting from different arrangements of scan directions and rotation axes for a 180° 2D Sick LMS200. This influential work studied the non-homogeneous distribution of range measurements of RSBLs by proposing a qualitative illustration of measured points on both a simulated hollow sphere surrounding the 3D scanner as well as on actual scans from representative environments. In [39], a subsampling method for RSBLs aimed at improving the homogeneity of measurements on the hollow sphere. Alismail and Browning [24] used a synthetic hollow cuboid with the RSBL at its center for quantitative assessment of the scanning pattern for calibration purposes. Furthermore, Schubert et al. [30] aimed at optimizing the alignment of a 2D rangefinder with respect to the additional DOF. With this purpose, they claim that a cost function can be computed from the density distribution of points on the hollow sphere. Regarding RMBL sensors, Neumann et al. [12] offered a comparison between several high-end MBLs and customized RMBLs by using both a qualitative analysis of example scenes and quantitative performance indices that are representative of particular device specifications, such as scanning time, data rate, and average point density on the sphere.

### 2.3. Our Approach

In this work, we focus on the analysis of measurement distribution of scan data, so calibration aspects fall outside of the scope of the paper. The analysis of spatial data distribution is especially interesting for the emergent RMBL configuration, since the vertical and horizontal resolutions of the resulting measurements is uneven due to the overlapping of multiple beams during rotation.

The review of published works indicates that commonly used indicators of spatial data distribution, such as Ripley’s *K* function [16], have not been considered in the analysis of 3D scanning patterns. The *K* function is useful to investigate the homogeneity of points for different ranges of distances, which has been applied to identify clusters from actual 3D scans of natural terrain [40]. Interestingly, a recent definition of the *K* function for spherical point-pattern analysis on planetary-scale distributions [17] allows that the use of this indicator can be extended to analyze scan patterns on the hollow sphere.

Thus, we propose to adapt spherical formulation of the *K* function [17] within an simulation-based analysis approach, both qualitative and quantitative, of the scan patterns produced by 3D lidar data. This analysis is applied to study general scan patterns produced by an ideal full-sphere RMBL based on the VLP-16 characteristics [33]. The proposed analysis approach allows comparing the ideal RMBL sensor with other 3D scan alternatives (i.e., SMBL and MBL). Besides, it can also be used to analyze a particular sensor implementation and to contrast it with the ideal RMBL. The latter point is illustrated through a case study with the *Velomotion-16* RMBL. This new sensor is an addition to the few RMBLs that have been reported recently [12,13,14,31].

## 3. Rotation of a Multi-Beam Lidar Sensor

This section provides a general definition for an RMBL whose rotation axis is parallel to one of the MBL frame axes and presents the computation of Cartesian point clouds. Without loss of generality, the VLP-16 [33] will be considered in this work for the addition of a rotation mechanism. This MBL sensor is especially suitable to build an RMBL on account of its more accessible cost, lighter weight, symmetric FOV, and compact size. Nevertheless, the following definitions could be extended to any MBL.

**Figure 1:**
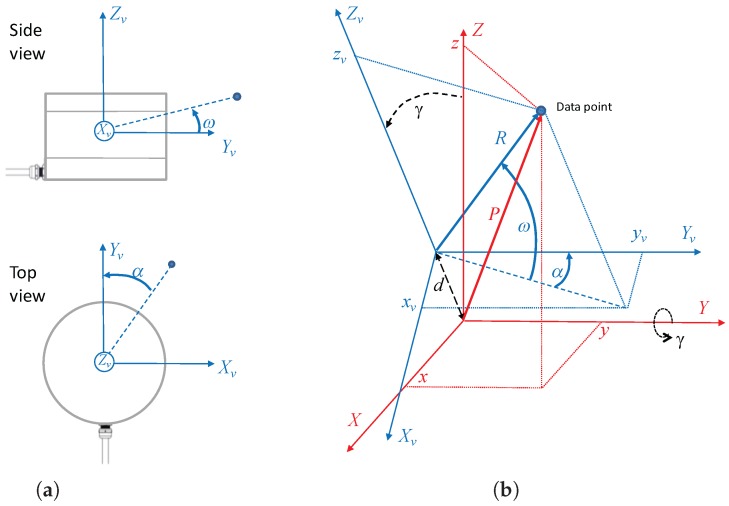
Reference frames and data point coordinates: (**a**) Velodyne VLP-16 sensor; and (**b**) RMBL based on a VLP-16. The VLP-16 local frame is represented in blue; the RMBL local frame is represented in red.

The local frame $X_{v}Y_{v}Z_{v}$ of the VLP-16 is shown in Figure 1a. This frame has its origin in the optical center, with the $Y_{v}$ axis in the forward direction and $Z_{v}$ pointing upwards. The VLP-16 scans points in spherical coordinates (R,ω,α). With this information, Cartesian coordinates $\left(x_{v},y_{v},z_{v}\right)$ can be obtained for each measured point:

(1) $$\begin{array}{ccc} x_{v} & = & R\cos(\omega )\sin(\alpha ), \end{array}$$

(2) $$\begin{array}{ccc} y_{v} & = & R\cos(\omega )\cos(\alpha ), \end{array}$$

(3) $$\begin{array}{ccc} z_{v} & = & R\sin(\omega ). \end{array}$$

The local frame XYZ of the RMBL resulting from the addition of a rotating mechanism (i.e., spinning or tilting) to the VLP-16 is illustrated in Figure 1b. When the rotation angle γ is null, $Z_{v}$ is aligned with *Z*, and $X_{v}$ and $Y_{v}$ are parallel to *X* and *Y*, respectively. Let us consider that the rotation axis is parallel to one of the VLP-16 axes; in this case, the rotation axis is *Y*. It should be noted that rotation about the *Y* and *X* axes would be similar, as the VLP-16 has a 360° horizontal FOV, whereas rotation about *Z* would be pointless as this is redundant with the spinning motion of the multi-beam transceivers. Furthermore, in practice, the rotation axis should be at some small distance *d* below the VLP-16 for the sake of compactness and shadow avoidance.

Then, Cartesian coordinates (x,y,z) of data points in the RMBL frame can be computed as:

(4) $$\begin{array}{ccc} x & = & R(\cos(\omega )\sin(\alpha )\cos(\gamma )+\sin(\omega )\sin(\gamma ))+d\sin(\gamma ), \end{array}$$

(5) $$\begin{array}{ccc} y & = & R\cos(\omega )\cos(\alpha ), \end{array}$$

(6) $$\begin{array}{ccc} z & = & R(\sin(\omega )\cos(\gamma )-\cos(\omega )\sin(\alpha )\sin(\gamma ))+d\cos(\gamma ). \end{array}$$

## 4. Analysis Methodology

This section proposes a simulation-based methodology to analyze, both qualitatively and quantitatively, the spatial distribution of laser beams in 3D lidars. First, the simulation of sensor points on a hollow sphere is defined. Qualitative analysis is done from a visualization of point patterns projected on the sphere and also on orthogonal planes. For a quantitative analysis, two spatial descriptive statistics are considered: sampling density and homogeneity. We propose adapting a spherical extension of Ripley’s *K* function to evaluate scanning homogeneity.

### 4.1. Numerical Simulation

The proposed methodology considers the set of points computed by simulating a scan from a sensor that is placed at the center of a virtual hollow sphere [15,30]. This structure allows analyzing the homogeneity and the beam density in all directions around the sensor. By using a sphere, the analysis depends exclusively on sensor characteristics and is independent of the orientation and distance of the target surface (e.g., as in planar targets). Moreover, the azimuth and elevation of the points in the sphere are independent of the sphere radius.

To analyze the general patterns produced by different scan configurations, Section 5 will consider ideal sensors that are independent of particular device considerations. To generate a complete sphere, an ideal RMBL sensor based on a VLP-16 lidar can be simulated by considering a constant angular velocity of the tilt motion from $\gamma =0^{\circ }$ to $\gamma =180^{\circ }$. Different angular velocities correspond to different scan resolutions for the additional DOF. For the sake of simplicity, it will also be assumed that the sphere radius is large enough to make the deviation between the center of the sphere (i.e., the RMBL’s origin) and its optical center negligible (i.e., d≈0); thus, all ranges *R* coincide with the sphere radius and Equations (1)–(3) can be applied. Moreover, as points are intended to represent beam directions, no noise is considered in the simulations. Besides, for generalization, no shadows or other FOV limitations due to a particular mechanism are considered.

For a qualitative analysis, it is also interesting to consider how the angular distribution of points on the sphere would be translated onto planar surfaces in a synthetic environment. In the proposed analysis, the sensors is placed at a height of 1.5 m over a ground plane that is parallel to the local XY plane and at 10 m from planes (representing walls) that are parallel to YZ and XZ planes. We have preferred this synthetic configuration rather than placing the sensor at the center of a 10 m × 10 m cube [24] because it is more representative of ground-based lidar applications.

### 4.2. Sampling Density

Sampling density for different scanning angles [15,24] can be represented as a 2D histogram on the sphere. With this aim, the sphere surface is partitioned with a triangular mesh obtained by recursive icosahedron sphere tessellation [41]. In this work, the sphere surface has been partitioned into 5120 bins.

### 4.3. Spatial Distribution Analysis of Scan Data with the K Function

A set of points in the plane is considered homogeneous if the same number of points occurs in any circular region of a given area. A common approach to analyze data homogeneity is Ripley’s *K* function [16]. The comparison of the *K* functions for complete spatial randomness (CSR) and for a given point set allows determining whether points have a random, dispersed or clustered distribution over a range of distances.

In this work, we need to evaluate homogeneity of points on a sphere. With this purpose, we adopt the *K* function proposed in [17], which modifies the planar *K* function and the corresponding CSR reference for spherical surfaces.

The *K* function for spherical CSR is given by:

(7) $$K_{csr}\left(r/R\right)=2\pi R^{2}\left(1-\cos\left(r/R\right)\right)$$

where *R* is the radius of the sphere and *r* is the great-circle distance, with r/R∈[0,π].

Given a set of *n* sphere points $p_{1},\ldots ,\;p_{n}$, the estimation of the *K* function can be expressed as:

(8) $$\hat{K}\left(r/R\right)=\frac{8\pi R^{2}}{n(n-1)}\sum_{i=1}^{n}\sum_{j=i+1}^{n}I\left(\theta \left(p_{i},p_{j}\right)\leq r/R\right),$$

where $\theta (p_{i},p_{j})$) represents the angle corresponding to the great-circle distance between $p_{i}$ and $p_{j}$, and I(·) is the indicator function.

Equation (8) is a modification with respect to [17] that accounts for the reciprocity of great circle distances between pairs of points, so the number of required computations is halved. Then, in order to apply this concept to 3D sensor homogeneity assessment, this equation is applied to the set of simulated scan points for discretized angle increments Δr/R in the interval [0,π]. For an RMBL based on a VLP-16, a value of Δr/R=π/151 is appropriate so that all bins contain a representative number of samples.

## 5. Analysis of Ideal RMBL Scanning Patterns and Comparison with Other 3D Configurations

The methodology defined in Section 4 has been applied to an ideal full-sphere VLP-16-based RMBL, which is compared with alternative 3D lidar configurations.

### 5.1. Qualitative Analysis

**Figure 2:**
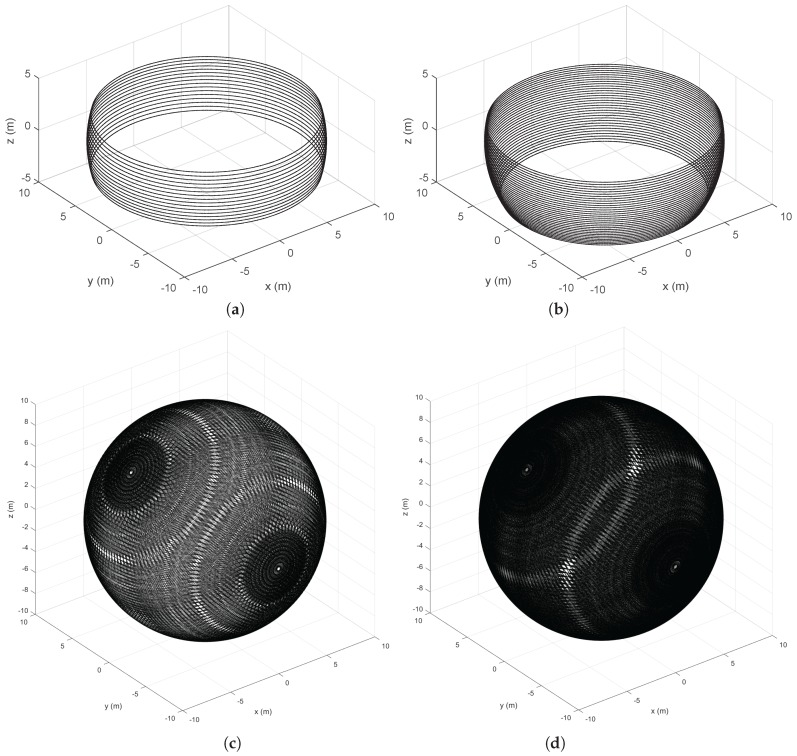
Representation of lidar beams as sphere points: (**a**) VLP-16; (**b**) HDL-32; (**c**) RMBL with tilting speed of $120^{\circ }$/s (29 frames); and (**d**) RMBL with tilting speed of $50^{\circ }$/s (71 frames).

**Figure 3:**
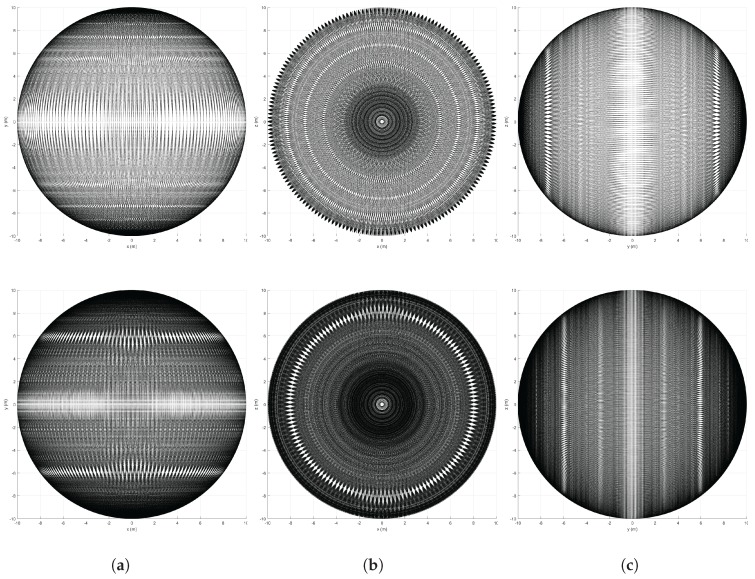
Different views of the sphere points given by the RMBL with tilting speed of $120^{\circ }$/s (top) and $50^{\circ }$/s (bottom): XY plane (**a**); XZ plane (**b**); and YZ plane (**c**).

A visualization of sphere points given by the 3D scanners is offered in Figure 2. These points are shown in local sensor coordinates for a sphere of radius R=10 m. Figure 2a,b illustrates the inhomogeneous beam pattern of the Velodyne sensors as well as their differences in vertical resolution and FOV. Moreover, Figure 2c,d shows points from RMBLs with different tilt speeds (i.e., different vertical resolutions). Points are distributed over the complete sphere but patterns due to the combination of the VLP-16 beams and the additional rotation are visible, especially in the lower resolution case. These patterns can also be appreciated on the lateral views of the spheres shown in Figure 3. Furthermore, some pattern distortion is appreciable in the central vertical strip in Figure 3a,b, which corresponds to a range of $\left[-15^{\circ },+15^{\circ }\right]$ around the extremes of the tilting motion. This can be explained because the eight VLP-16 transducers with positive ω elevation values when $\gamma =0^{\circ }$ are overlapped with the eight transducers with negative ω when $\gamma =180^{\circ }$, and vice versa.

**Figure 4:**
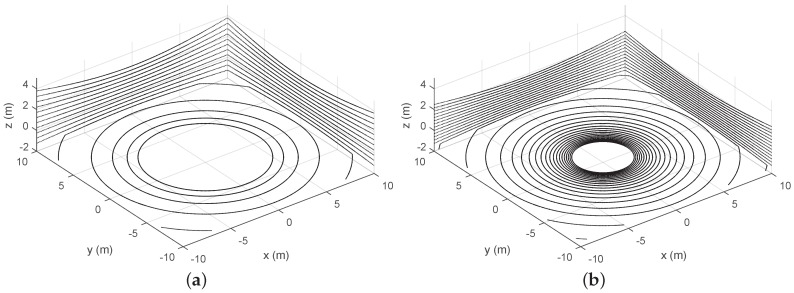

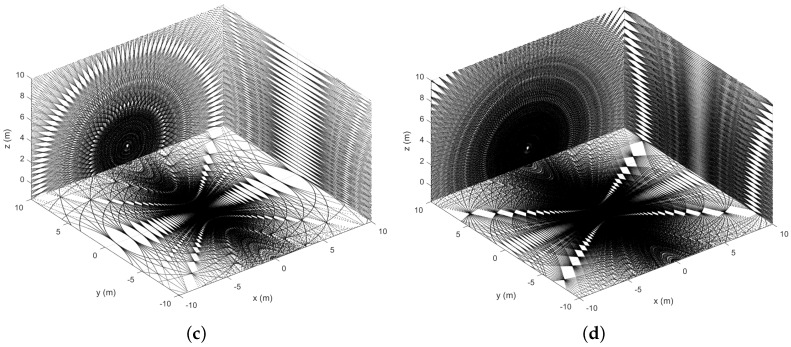
Orthogonal plane points of lidar sensors: (**a**) VLP-16; (**b**) HDL-32; (**c**) RMBL with tilting speed of 120°/s (29 frames); and (**d**) RMBL with tilting speed of 50°/s (71 frames).

The translation of scan points onto planar surfaces in a synthetic environment is illustrated in Figure 4. The figure shows how the VLP-16 provides scarce information about the ground. This is improved in the HDL-32, but the maximum height of wall points is reduced. The RMBL offers denser data and a wider FOV for both ground and walls. Besides, it can be observed that RMBL point patterns on the target planes depend on the sensor orientation. Thus, the wall perpendicular to the *X* axis is scanned with a higher density on the sides (i.e., similar to a good peripheral vision), whereas the wall perpendicular to the *X* axis is sampled with a higher density in the area that is close to the tilting axis. These differences should be considered when deciding on the sensor orientation with respect to the target surfaces in a particular application. Moreover, Figure 4d shows that a pattern of blind spots remains despite increasing the vertical scan resolution.

### 5.2. Sampling Density

**Figure 5:**
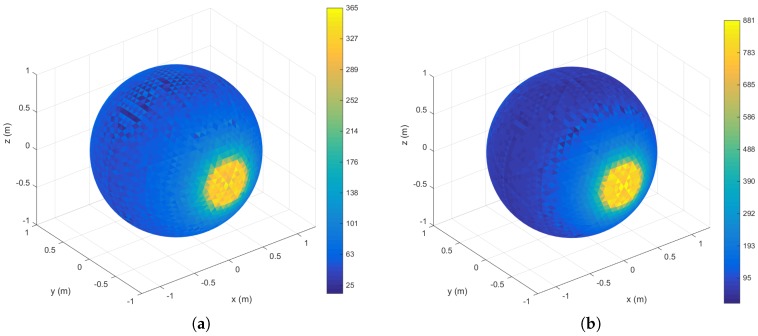
2D histogram of beam density on the sphere: (**a**) RMBL with tilting speed of $120^{\circ }$/s (29 frames); and (**b**) RMBL with tilting speed of $50^{\circ }$/s (71 frames). Color bars indicate points/bin.

Beam density histograms for the RMBL with tilting speeds of $120^{\circ }$/s and $50^{\circ }$/s are presented in Figure 5. For each case, the color scale has been normalized with respect to the maximum and minimum number of points per bin. The density increases notably in the polar regions (i.e., around the intersection with the rotation axis), which is a common trait with rotating 2D scanners [39]. The figure reflects that the slower scan offers more density, but also that the density distributions are similar regardless of tilting speed. However, a slightly darker and wider equator band in Figure 5b would indicate a lower density in relation to the poles for the slower scan, which can be attributed to greater polar oversampling. The triangles with the maximum number of beams are in the polar regions, with 365 points for the $120^{\circ }$/s scan and 881 for $50^{\circ }$/s. In the lower resolution scan, the triangles with the minimum number of points (i.e., 25) lie around the equator band. When the tilt resolution is increased, the bins with fewer points (i.e., 95) are in two parallel bands closer to each polar region. These parallel dead zones are not completely eliminated by increasing tilt resolution, as can be seen in Figure 3. The dead zones can be explained by the elevation gap between the VLP-16 transducers.

**Figure 6:**
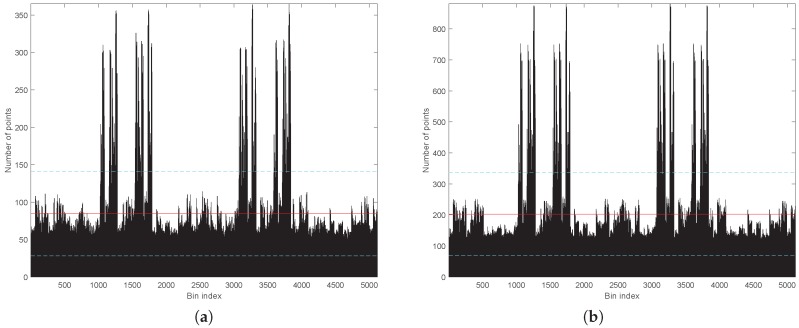
Beam density histogram: (**a**) RMBL with tilting speed of $120^{\circ }$/s (29 frames); and (**b**) RMBL with tilting speed of $50^{\circ }$/s (71 frames). The red solid lines indicate the mean value and the blue dotted lines are the standard deviation.

The same data is represented in Figure 6 in a classical histogram graph. This figure indicates that the number of points is quite homogeneous with the exception of two groups of peaks that correspond to the polar regions. Again, the shapes of the histograms for both tilting speeds (i.e., elevation resolutions) are very similar. For the $120^{\circ }$/s speed, the mean is 10.48 points/deg^2^ (84.47 points/bin) with a standard deviation of 6.98 points/deg^2^ (56.24 points/bin); for $50^{\circ }$/s, the mean value is 25.16 points/deg^2^ (202.73 points/bin) with a standard deviation of 16.62 points/deg^2^ (133.92 points/bin).

### 5.3. Spatial Distribution Analysis of Scan Data with the K Function

**Figure 7:**
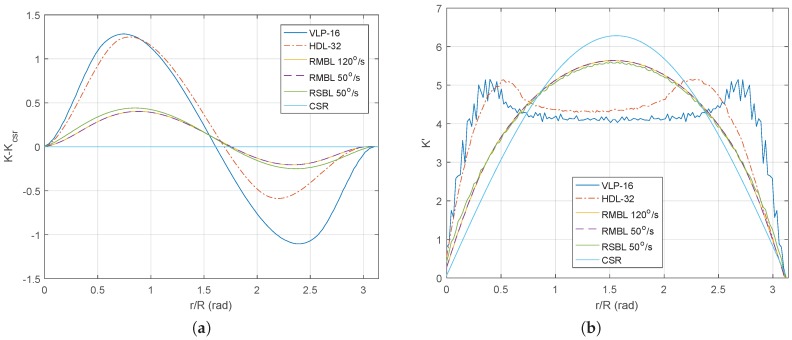
Homogeneity analysis of point patterns on the sphere: (**a**) deviation of $\hat{K}$ with respect to the reference $K_{csr}$; and (**b**) first derivative of $K_{csr}$ and $\hat{K}$.

The spherical *K* function has been computed for the hollow sphere data patterns that correspond to different MBL, RSBL, and RMBL sensor configurations. Two different representations of the resulting functions are given in Figure 7: the deviation of $\hat{K}$ with respect to the reference $K_{csr}$ and the first derivative of $\hat{K}$.

The deviation of $\hat{K}$ with respect to the CSR reference value $K_{csr}$ in Equation (7) is represented in Figure 7a for different great-circle distances *r* normalized by the sphere radius *R*. In particular, Equation (8) has been used to compute $\hat{K}$ for the VLP-16, the HDL-32, two different tilting speeds of a VLP-16 based RMBL, and a generic RSBL with the same horizontal resolution as the VLP-16 and a tilt speed of $50^{\circ }$/s. It can be appreciated that the spherical point pattern homogeneity for the RMBL is independent of the tilting speed, as the curves for $120^{\circ }$/s and $50^{\circ }$/s are overlapped.

Positive values in Figure 7a indicate clustering, i.e., that the average number of neighbor points for that particular range of evaluation distances is higher than the average for the whole distribution, whereas negative values indicate dispersion. The graph indicates that clustering is similar for the VLP-16 and HDL-32 sensors, while dispersion is larger for the VLP-16. In the VLP-16, the maximum values for both clustering and dispersion are similar, as opposed to the HDL-32, where clustering is significantly larger than dispersion. This difference can be explained by the asymmetrical FOV of the HDL-32 (see Figure 2a,b), as samples from the lower beams (i.e., the lower values of the *Z* coordinate) are denser. Correspondingly, the higher sampling density of the poles in the RMBL sensor (as shown in Figure 5) provokes that clustering is larger than dispersion, which also happens in the RSBL. All in all, the curves for the RMBL are clearly closer to $K_{csr}$ than the Velodyne sensors and offer some improvement over the RSBL.

Figure 7b presents the derivative of $\hat{K}$ with respect to the normalized great-circle distance r/R. This graph is interesting because its values for a given distance represent observed frequencies of points separated by that particular distance. The discretization of the r/R axis in 151 sections explains the noise-like aspect of the VLP-16 curve (i.e., the one with the smallest number of samples). The reference curve $K_{csr}$ is symmetrical with respect to the great-circle distance and reaches its maximum at the central value (i.e., for points at about a distance of 90°). This central peak value does not appear in the VLP-16 and HDL-32 curves, where large portions of the sphere are not sampled. Furthermore, some asymmetry is evident for the HDL-32, which is consistent with its asymmetric FOV. The tilting sensors give results that are much closer to the $K_{csr}$, with a slight advantage of the RMBL with respect to the RSBL.

## 6. Implementation of a Portable Tilting Mechanism for a Velodyne VLP-16

**Figure 8:**
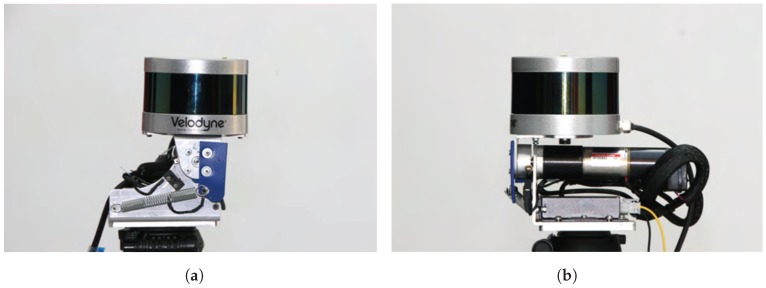
Views of the Velomotion-16 RMBL sensor: (**a**) side; and (**b**) front.

The analysis methodology presented in Section 4 can be used to assess the scan measurement distribution of a real MBRL device and to establish a comparison with the ideal full-sphere values obtained in Section 5. An implementation of an RMBL consisting on a tilting multi-beam laser scanner has been developed in this work. This new device, named *Velomotion-16*, has been designed as a light portable platform based on the Velodyne VLP-16 scanner (see Figure 8).

### 6.1. Velomotion-16 System Description

**Figure 9:**
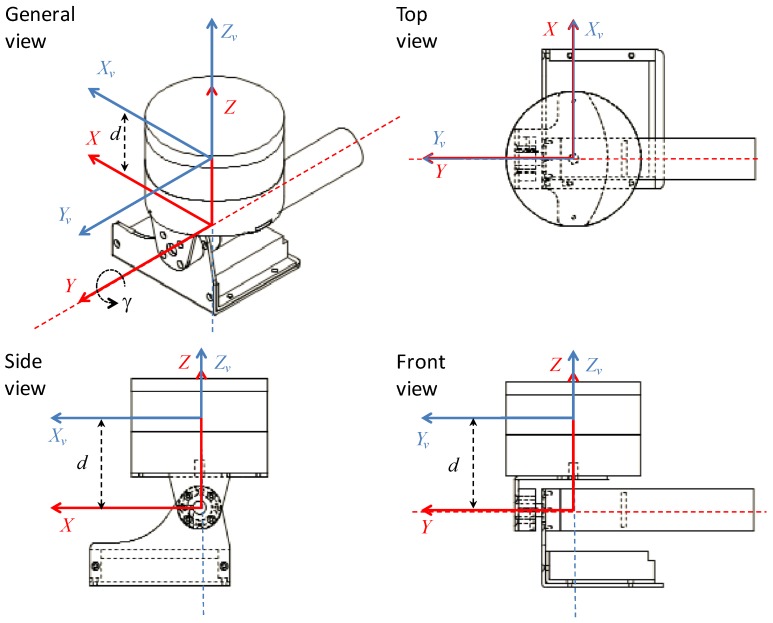
*Velomotion-16* reference frames and tilting parameters. The VLP-16 local frame is represented in blue; the *Velomotion-16* local frame is represented in red.

Several views of the *Velomotion-16* sensor design are presented in Figure 9. This figure shows the frames for VLP-16 frame and the *Velomotion-16* using the same notation as in Figure 1, including the tilting angle γ and the relative distance between the rotation mechanism and the optical center, which is d=6 cm.

The main specifications of the tilting platform are presented in Table 3, where some parameters are inherited from the constituent VLP-16 sensor. In this device, there is a mechanical limitation regarding the additional DOF, which is in the range $\left[-45^{\circ },0^{\circ }\right]$. This means that the vertical FOV is asymmetrical with respect to the horizontal plane, as it is $\left[-60^{\circ },15^{\circ }\right]$ in the forward direction and $\left[-15^{\circ },60^{\circ }\right]$ backwards.

The range of Velomotion-16 is inherited from that of the VLP-16, but it is affected by a positive offset not greater than *d*. The actual limits of the sensing range depend on (α,ω,γ). Given that a range measurement *P* (see Figure 1) is:

(9) $$P=\sqrt{x^{2}+y^{2}+z^{2}},$$

then, the actual minimum and maximum range values for *P* can be computed using Equations (4)–(6) with R=1 m and R=100 m, respectively.

From a mechanical standpoint, the portable tilting platform consists of two L-shaped links with a rotational joint. The base link has been designed to accommodate the controller card and the motor-gear-break set and includes two switches to restrict the displacement. The VLP-16 support link has been designed to be lightweight, to achieve a small *d* value, and to make the *Y* and the $Y_{v}$ axes parallel, as in Figure 1. A cylindrical coupling piece joins the output axis from the reduction gear with the VLP-16 support. Furthermore, a spring avoids the clearance between the base link and the VLP-16 support.

**Figure 10:**
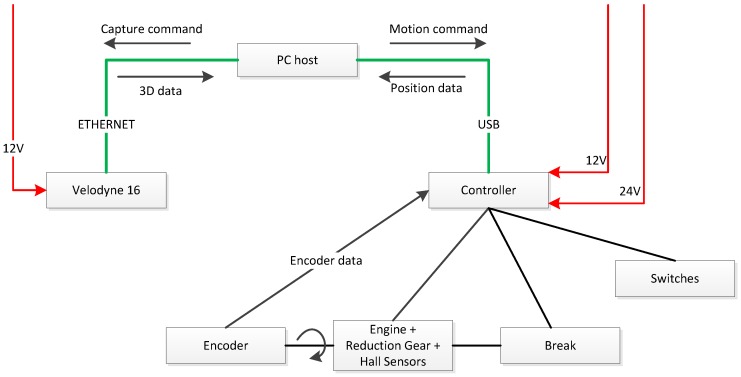
Velomotion-16 system architecture.

A general overview of the system architecture is shown in Figure 10. The tilting motion is achieved by an EC brushless motor with encoder and brake and an EPOS2 controller, both by Maxon (Sachseln, Switzerland). Two 12 V batteries are used to provide 12 V power to the system (including the VLP-16) and 24 V to the brake. Motion control is performed through a trapezoidal profile in which speed, acceleration and deceleration can be specified by the user to produce a particular scanning resolution. The fastest scan is achieved by setting the tilting speed to 56.25°/s, which corresponds to eight VLP-16 scans. Conversely, high density scans can be obtained by programming slower tilt speeds, which can be as low as 0.05°/s.

As illustrated in Figure 10, the PC host sends capture commands and receives 3D data as Robot Operating System (ROS) messages from the VLP-16 via Ethernet. Moreover, the PC sends the goal position of the motion profile and receives the current angle from the tilting platform through a USB connection. A ROS driver has been developed to synchronize consecutive VLP-16 scans with the corresponding tilt angles in order to generate a dense point cloud.

### 6.2. Analysis of the Scan Measurement Distribution for Velomotion-16

**Figure 11:**
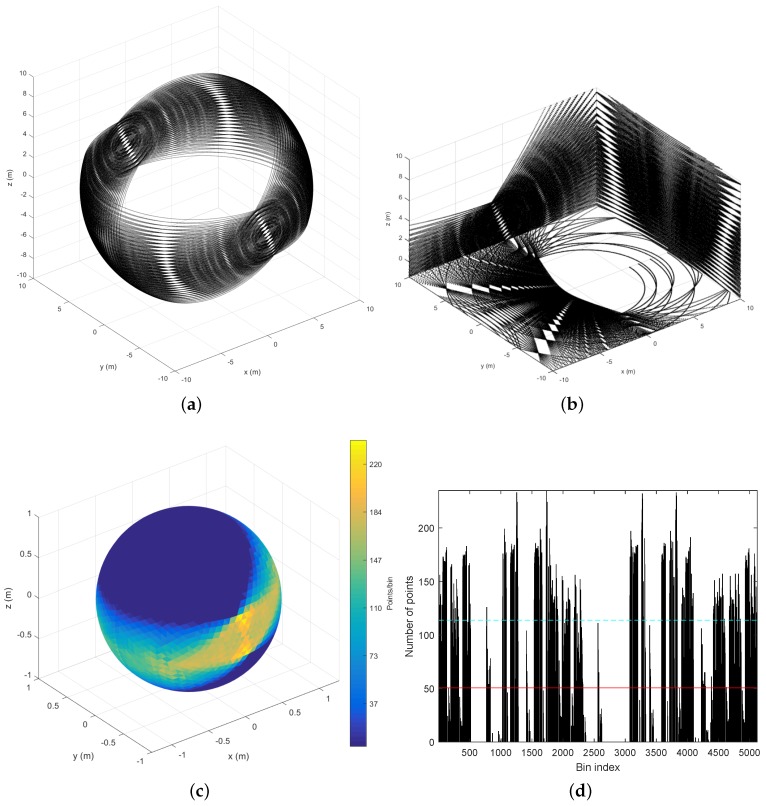

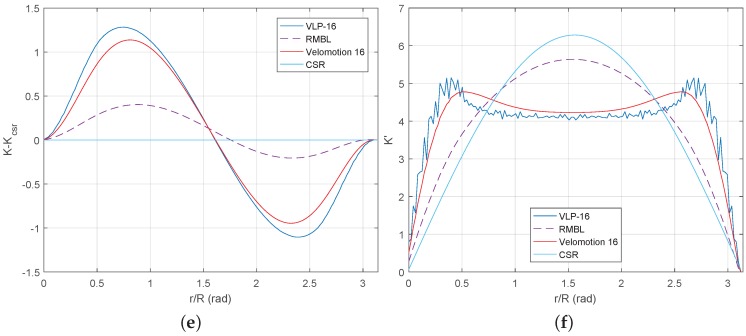
Analysis of 3D scan measurement distribution for *Velomotion-16* with tilting speed of 50°/s (17 frames): (**a**) hollow sphere points; (**b**) orthogonal plane points; (**c**) point density on the sphere (dark blue means no measurements); (**d**) point density as a histogram; (**e**) homogeneity analysis as $\hat{K}-K_{csr}$; and (**f**) homogeneity analysis as the first derivative of $\hat{K}$.

The effects on the scan measurement distribution produced by a particular sensor construction can be identified by applying the proposed analysis methodology. This is illustrated in Figure 11 for Velomotion-16, which has a limited tilting range. The results in the figure correspond to a tilting speed of $50^{\circ }$/s.

The complete FOV originated by the tilting range, which can be clearly appreciated in Figure 11a, has a downwards orientation in the negative direction of the *X* axis (i.e., backwards). The hollow sphere patterns are similar to those of the ideal sensor with the exception of the scan lines in the extremes of the tilt range, which are sparser because they are not overlapped. This sparseness can be appreciated in the forward floor plane points of Figure 11b. Orthogonal plane points also reveal the efficacy of Velomotion-16 to scan ground points in the backward direction, whereas the forward direction is more appropriate to capture higher vertical structures.

The point density histograms in Figure 11c,d also indicate unscanned regions as well as a slighter scan density in the extremes of the tilt range. The mean point density has been reduced from 25.16 points/deg^2^ (202.73 points/bin) in the ideal full-sphere to 6.29 points/deg^2^ (50.68 points/bin) in the Velomotion-16 sensor. Similarly, the standard deviation has changed from 16.62 points/deg^2^ (133.92 points/bin) to 7.85 points/deg^2^ (63.22 points/bin).

As for the *K* function, the difference of the Velomotion-16 with respect the CSR value (shown in Figure 11e) clearly improves homogeneity with respect to the original VLP-16 sensor, in both clustering and dispersion. However, the limited tilt range of the Velomotion-16 causes that the *K* function is closer to the VLP-16 than to the ideal RMBL. This homogeneity results can also be appreciated in the first derivative representation of the estimated *K* function seen in Figure 11f).

## 7. Discussion of Example Scans

**Figure 12:**
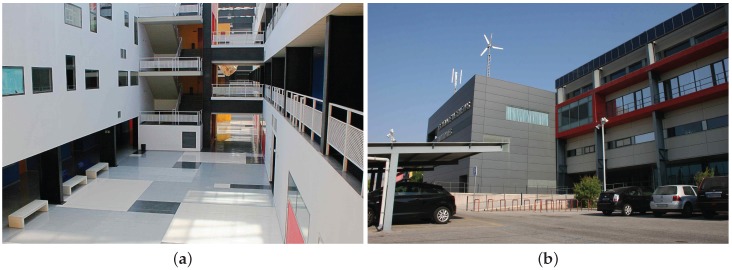

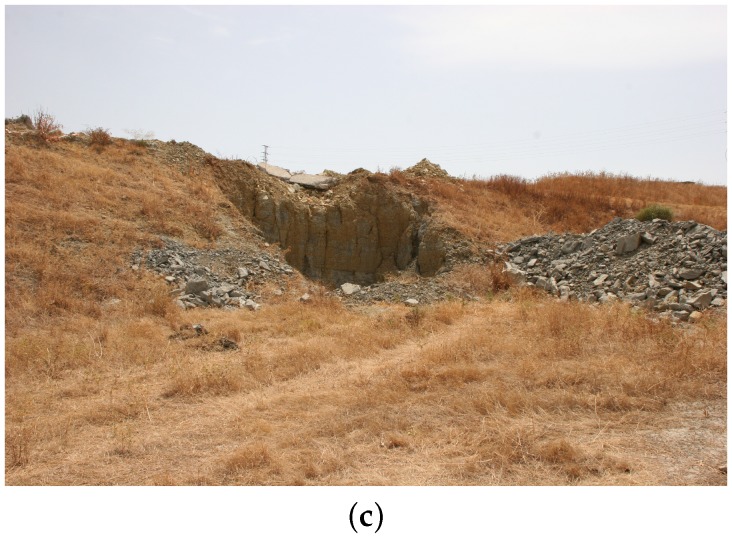
Photos of experimental scenes: (**a**) building hall (indoor); (**b**) parking area (urban); and (**c**) quarry (terrain).

The purpose of this section is to illustrate 3D lidar data from actual scans obtained in three representative scenes of indoor and outdoor environments: a building hall, an outdoor parking area in a urban setting, and a quarry with irregular terrain, respectively (see Figure 12). Actual scans have been obtained by Velodyne’s VLP-16 and HDL-32 MBLs as well as for the *Velomotion-16* RMBL with two different tilting speeds (i.e., vertical resolutions): the fastest scan speed given by the sensor (i.e., 56.25°/s) and a slow high resolution speed of 1.07°/s. The sensors have been placed on a tripod at a height of about 1.2 m.

Table 4 summarizes sensor performance by presenting the scan time for each case, as well as the resulting number of points for each scene. The total number of points given by the *Velomotion-16* is substantially larger in the indoor environment due to out-of-range measurements in the urban environment. This difference is not so important in the Velodyne sensors, as their FOV is very limited in the upwards direction. The fast *Velomotion-16* requires 0.8 s (i.e., eight times the Velodyne scan time) to capture eight VLP-16 scans, which produces a greater number of points (i.e., 7.21 times and 7.86 times, for the indoor and urban scenes, respectively) than a single VLP-16 scan as well as a wider FOV. Furthermore, the fast *Velomotion-16* also improves the number of points and the FOV of the HDL-32, which is a considerably more expensive sensor. By adjusting the *Velomotion-16* γ speed to a slower value, the resulting number of points, and the subsequent data density, can be greatly improved, as indicated by the numbers given by the slow case in the table.

**Figure 13:**
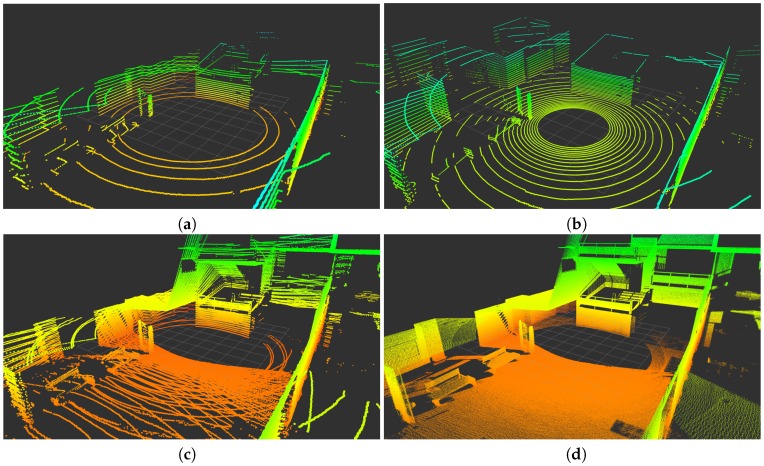
Scans from an indoor scene: (**a**) VLP-16; (**b**) HDL-32; (**c**) Fast *Velomotion-16*; and (**d**) Slow *Velomotion-16*.

**Figure 14:**
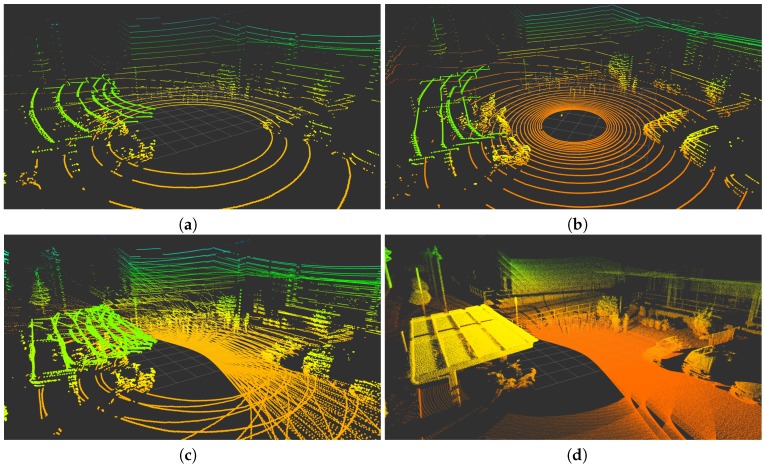
Scans from the urban scene: (**a**) VLP-16; (**b**) HDL-32; (**c**) Fast *Velomotion-16*; and (**d**) Slow *Velomotion-16*.

**Figure 15:**
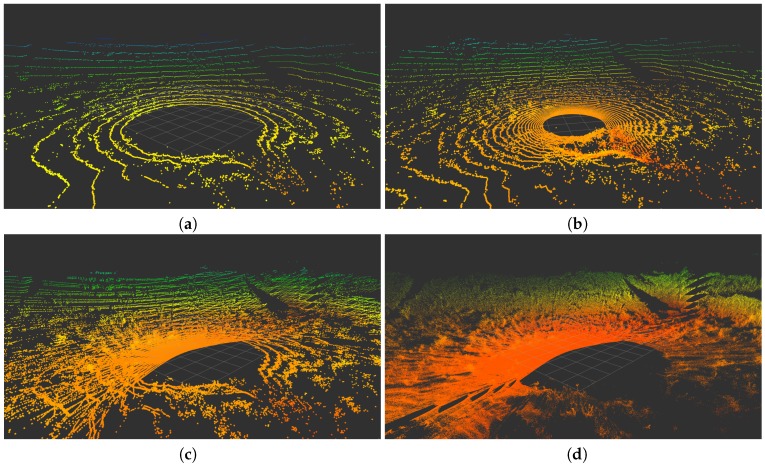
Scans from an outdoor terrain scene: (**a**) VLP-16; (**b**) HDL-32; (**c**) Fast *Velomotion-16*; and (**d**) Slow *Velomotion-16*.

The results for these experiments are presented in Figure 13, Figure 14 and Figure 15, where the color grading represents elevation. These illustrations confirm the improvement in data density and FOV provided by the *Velomotion-16* with respect to both Velodyne lidars. The particular mechanical rotation limits of the *Velomotion-16* favor a denser resolution of the floor in the forward direction and a better measurement of higher objects in the backwards direction. Interestingly, even the fast *Velomotion-16* offers a high point density in the immediate floor area. In the quarry scene, the forward scan direction provides a detailed scan of the ground terrain and the excavated wall, especially in the slow scan.

As for the particular scan patterns presented in Figure 4, these can also be appreciated in the floor and vertical walls of these actual scans. In general, these patterns become difficult to identify when the resolution is increased in the fast *Velomotion-16*, but the pattern of small blind spots can be seen on the wall at the center-left part of Figure 13d, the floor at the bottom-right side of Figure 14d, and the bottom-left diagonal and the top-right area of Figure 15d.

## 8. Conclusions

In this work, we have addressed rotating multi-beam lidar (RMBL) sensors, a type of customized 3D rangefinders built by adding a rotation mechanism to a commercial multi-beam lidar (MBL). Recent published examples using VLP-16 (Puck), the most affordable and lightest MBL by Velodyne, indicate that the RMBL configuration has the potential to become a common solution to get low-cost, rapid, and full-3D high resolution scans, as has happened with customized rotating single-beam lidars (RSML) during the last fifteen years. However, contrary to RSBLs, the additional DOF in RMBLs causes multiple scan beams to overlap creating complex scanning patterns.

Particularly, we have proposed a simulation-based methodology to analyze 3D scanning patterns, which has been applied to investigate the complex scan measurement distribution produced by the RMBL configuration. With this major purpose, novel contributions offered in the paper include the following: (i) the adaption of a recent spherical reformulation of Ripley’s *K* function to assess 3D sensor data distribution on hollow sphere simulations; (ii) a comparison, both qualitative and quantitative, of scan patterns produced by an ideal RMBL based on a Velodyne VLP-16 (Puck) and those of other 3D scan alternatives (i.e., rotating 2D lidar and MBL); and (iii) a discussion of experimental scans from three representative environments obtained from a new RMBL implementation consisting of a portable tilting platform for VLP-16 scanners, which have been compared with the VLP-16 and HDL-32E MBLs.

Qualitative analysis evidences particular sampling patterns provoked by the RMBL configuration. Most of these patterns are difficult to appreciate when the resolution of the additional rotation is increased with slower scans. Nevertheless, rows of characteristic small blind spots remain visible even with high resolution rotation. Apart from that, similarly to RSBLs, the measurement density has focal points in the rotation axis, which has to be considered when placing the sensor for a particular application.

Besides, the analysis of the spatial distribution of scan measurements with the spherical *K* function indicates that homogeneity is independent of the rotation speed. However, it has been observed that the *K* function deviates from complete spatial randomness due to unsampled regions, such as those resulting from the limited field of view (FOV) of MBLs, as well as by poles (or focal points) in RMBLs and RSBLs. The comparison of *K* function estimations between ideal full-sphere RMBLs and an equivalent RSBL yields similar results, with a slight advantage of the multi-beam based case.

The *Velomotion-16* sensor has been presented as case study to discuss actual scans from representative scenes as well as an illustration of the use of the proposed methodology to analyze the scan measurement distribution of an actual sensor with a limited tilt range. Thus, experimental example scans obtained by a VLP-16 on a tilting mechanism have illustrated a practical implementation of the RMBL configuration. These scans have shown that even a fast tilt (in less than one second) of the VLP-16 provides an environment description that can be considerably richer (both in FOV and number of points) than that of the HDL-32. A much higher level of detail can be appreciated in scans taken in less than a one-minute span.

Given the significant difference in price between the VLP-16 and the HDL-32 (not to mention other high-end 3D lidars), these results support the feasibility of customized RMBLs based on the least expensive MBLs (like the VLP-16) in applications demanding affordable and compact high-resolution point clouds without the FOV and vertical resolution limitations of commercial MBLs. The use of this type of sensor for robotic mapping can be done with stop-and-go scans or with continuous tilting, but the latter requires a more complicated registration process [13]. Furthermore, other future applications could benefit from the availability of a portable and affordable sensor producing high-resolution point clouds with no FOV limitations in a stop-and-go fashion. These potential applications could include: intelligent surveillance of public and private spaces; modeling and progress tracking in construction sites; modeling of caves, tunnels or narrow spaces in speleology, archeology, mining, and search and rescue; and body imaging for medicine, prosthetics, textile tailoring and other applications.

Further potentially interesting aspects include an analysis of accuracy and precision of the *Velomotion-16* device. We are investigating these aspects in ongoing studies. Moreover, it will be interesting to test the applicability of calibration methods devised for 2D lidar with a rotation mechanism (e.g., [23]) when applied to the rotation of multiple beams.

In the future, the proposed simulation methodology can be useful to assess the effect of other particular rotating mechanisms with respect to ideal measure distributions. Furthermore, it will also be interesting to analyze the effect of rotating other MBLs, since there is an increasingly active MBL market for new compact and cost-effective devices (e.g., the 32-beam Ultra Puck VLP-32C by Velodyne [11]).

## Figures and Tables

**Table 1:** Representative examples of customized lidar systems based on a commercial device with an extra DOF.

	Type	Device	Major Application
Batavia 2002 [7]	RSBL	Sick	Obstacle detection
Wulf 2003 [15]	RSBL	Sick LMS200	Density analysis
Weingarten 2006 [18]	RSBL	(2) Sick LMS200	Indoor scenario reconstruction
Dias 2006 [19]	RSBL	Sick LMS200	Device comparison
Sheh 2006 [8]	RSBL	Hokuyo URG-04LX	Sensor configuration analysis
Ueda 2006 [20]	RSBL	Hokuyo URG-04LX	Mapping
Yoshida 2010 [9]	RSBL	Hokuyo UTM-30LX	Mapping
Morales 2011 [21]	RSBL	Hokuyo UTM-30LX	Mapping and environment modeling
Xiao 2013 [22]	RSBL	Hokuyo UTM-30LX	Indoor mobile robot
Neumann 2014 [13]	RMBL	Velodyne HDL-64E	Underground mapping
Morales 2014 [23]	RSBL	Hokuyo UTM-30LX	Boresight calibration
Alismail 2015 [24]	RSBL	Hokuyo UTM-30LX-EX	Calibration for 3D mapping
An 2015 [25]	RSBL	Hokuyo URG-30LX	Plane extraction from indoor robot
Martinez 2015 [26]	RSBL	Hokuyo UTM-30LX-EX	UGV and UAV environment modeling
Özbay 2015 [27]	RSBL	Hokuyo UTM-30LX	UGV obstacle modeling
Moon 2015 [28]	RSBL	SICK LMS511-pro	Cargo ship modeling
Shaukat 2016 [29]	RSBL	Hokuyo UTM-30LX	RGB-D terrain modelling
Schubert 2016 [30]	RSBL	Hokuyo UTM-30LX	Robot mapping
Leingartner 2016 [31]	RMBL	Velodyne HDL-64E	Mapping
Neumann 2016 [12]	RSBL	Hokuyo UTM-30LX-EW	RMBL and MBL comparison
	RMBL	and Velodyne VLP-16
Kang 2016 [32]	RSBL	Hokuyo UTM-30LX	6 DOF calibration
Droeschel 2017 [10]	RSBL	Hokuyo UTM-30LX-EW	Robot mapping
Klamt 2017 [14]	RMBL	Velodyne VLP-16	Robot mapping

**Table 2:** Manufacturer specifications for the VLP-16 and HDL-32 sensors [33].

	VLP-16	HDL-32
Laser/detector pairs	16	32
Range	1 m to 100m	1 m to 70 m
Accuracy	±3 cm	± 2 cm
Data	Distance/Calibrated reflectivities	Distance/Calibrated reflectivities
Data Rate	300,000 points/s	700,000 points/s
Vertical FOV	$30^{\circ }:\left[-15^{\circ },+15^{\circ }\right]$	$41.3^{\circ }:\left[-30.67^{\circ },+10.67^{\circ }\right]$
Vertical Resolution	2.0°	1.33°
Horizontal FOV	360°	360°
Horizontal Resolution	0.1° to 0.4° (programmable)	0.08° to 0.35° (programmable)
Size	103 mm × 72 mm	85.3 mm × 149.9 mm
Weight	0.83 Kg	1.3 Kg

**Table 3:** Specifications of Velomotion-16, as used in the case study (VLP-16 device included). When values are inherited from the VLP-16, this is indicated.

Range	1 m to 100 m (VLP-16) + offset ≤d
Accuracy	±3 cm (VLP-16)
Data Rate	300,000 points/s (VLP-16)
*d*	6 cm
Tilting range	$\left[-45^{\circ },0^{\circ }\right]$
Tilting speed	0.05°/s to 56.25°/s (programmable)
Vertical FOV	$75^{\circ }:\left[-60^{\circ },+15^{\circ }\right]$ (forwards), $\left[-15^{\circ },60^{\circ }\right]$ (backwards)
Vertical Resolution	uneven
Mean vertical resolution	5.2° $\cdot 10^{-3}$ to 0.59° (programmable)
Horizontal FOV	360° (VLP-16)
Horizontal Resolution	0.1° to 0.4° (programmable) (VLP-16)
Size	105 mm width × 95 mm height × 165 mm depth
Weight	1.9 kg (+0.7 kg wires)

**Table 4:** Sensor performance in example scans.

	Scan Time (s)	γ Speed (°/s)	Indoor: Points	Urban: Points	Terrain: Points
*Velomotion-16* (slow)	42.05	1.07	11,348,103	6,833,873	6,269,304
*Velomotion-16* (fast)	0.80	56.25	201,851	158,351	125,237
VLP-16	0.10	-	27,998	20,141	16,954
HDL-32	0.10	-	68,080	58,875	51,362
